# CT classification model of pancreatic serous cystic neoplasms and mucinous cystic neoplasms based on a deep neural network

**DOI:** 10.1007/s00261-021-03230-5

**Published:** 2021-10-12

**Authors:** Rong Yang, Yizhou Chen, Guo Sa, Kangjie Li, Haigen Hu, Jie Zhou, Qiu Guan, Feng Chen

**Affiliations:** 1 Department of Radiology, The First Affiliated Hospital, Zhejiang University School of Medicine, #79 Qingchun Road, Hangzhou, 310003 Zhejiang Province P.R. China; 2 College of Computer Science and Technology, Zhejiang University of Technology, #288 Liuhe Road, Hangzhou, 310023 Zhejiang Province P.R. China; 3 Department of Pathology, The First Affiliated Hospital, Zhejiang University School of Medicine, #79 Qingchun Road, Hangzhou, 310003 Zhejiang Province P.R. China

**Keywords:** Pancreas, SCNs, MCNs, Computed tomography, Deep neural network

## Abstract

**Background:**

At present, numerous challenges exist in the diagnosis of pancreatic SCNs and MCNs. After the emergence of artificial intelligence (AI), many radiomics research methods have been applied to the identification of pancreatic SCNs and MCNs.

**Purpose:**

A deep neural network (DNN) model termed Multi-channel-Multiclassifier-Random Forest-ResNet (MMRF-ResNet) was constructed to provide an objective CT imaging basis for differential diagnosis between pancreatic serous cystic neoplasms (SCNs) and mucinous cystic neoplasms (MCNs).

**Materials and methods:**

This study is a retrospective analysis of pancreatic unenhanced and enhanced CT images in 63 patients with pancreatic SCNs and 47 patients with MCNs (3 of which were mucinous cystadenocarcinoma) confirmed by pathology from December 2010 to August 2016. Different image segmented methods (single-channel manual outline ROI image and multi-channel image), feature extraction methods (wavelet, LBP, HOG, GLCM, Gabor, ResNet, and AlexNet) and classifiers (KNN, Softmax, Bayes, random forest classifier, and Majority Voting rule method) are used to classify the nature of the lesion in each CT image (SCNs/MCNs). Then, the comparisons of classification results were made based on sensitivity, specificity, precision, accuracy, F1 score, and area under the receiver operating characteristic curve (AUC), with pathological results serving as the gold standard.

**Results:**

Multi-channel-ResNet (AUC 0.98) was superior to Manual-ResNet (AUC 0.91).CT image characteristics of lesions extracted by ResNet are more representative than wavelet, LBP, HOG, GLCM, Gabor, and AlexNet. Compared to the use of three classifiers alone and Majority Voting rule method, the use of the MMRF-ResNet model exhibits a better evaluation effect (AUC 0.96) for the classification of the pancreatic SCNs and MCNs.

**Conclusion:**

The CT image classification model MMRF-ResNet is an effective method to distinguish between pancreatic SCNs and MCNs.

**Graphic abstract:**

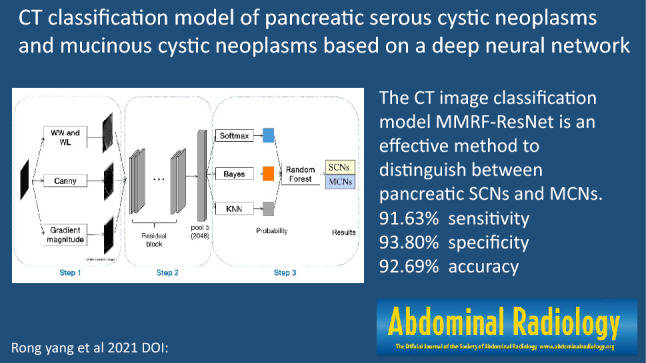

**Supplementary Information:**

The online version contains supplementary material available at 10.1007/s00261-021-03230-5.

## Introduction

**Fig. 1:**
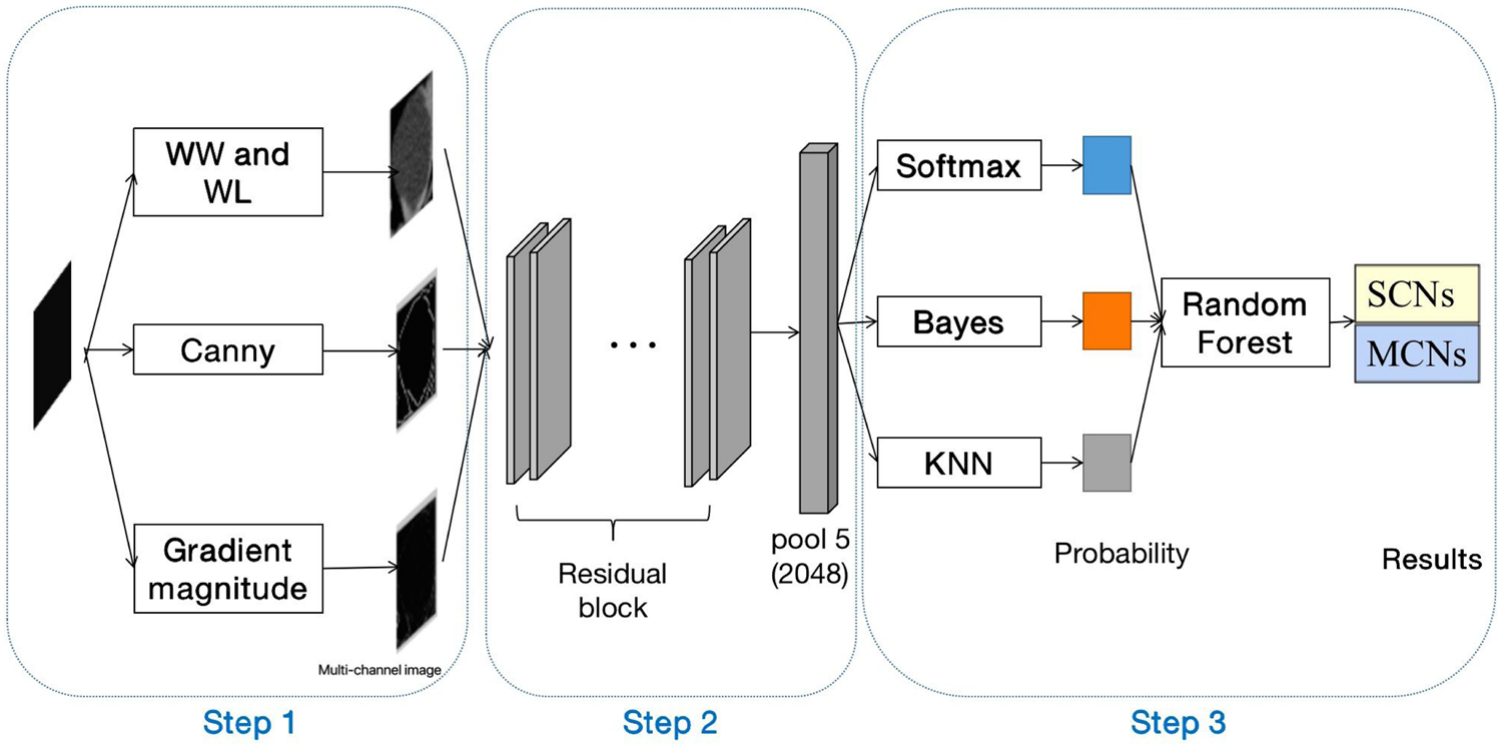
MMRF-ResNet classifier flow chart. Step 1: Image segmentation. Step 2: Feature extraction. Step 3: Classification. WW: Window width, WL: Window level, Canny: An efficient edge detection algorithm, Gradient magnitude: Magnitude of pixel gradient in the image. Gradient is a vector with direction and size, DNN: Deep Neural Network, Residual block and pool 5: Network structure in convolutional neural networks, Softmax, Bayes, KNN, Random forest: a type of classifier

Pancreatic serous cystic neoplasms (SCNs) and mucinous cystic neoplasms (MCNs) are rare types of pancreatic tumors. According to the WHO (2010) digestive system tumor classification guidelines, SCNs are benign tumors and MCNs are classified into low/intermediate/high-grade dysplasia and invasive carcinoma [1]. Most SCNs do not require surgery unless tumors grow significantly faster or aggressively, while MCNs require surgical treatment once they are found. Therefore, it is necessary to distinguish pancreatic SCNs from MCNs. After the emergence of artificial intelligence (AI), many radiomics research methods have been applied to the identification of pancreatic SCNs and MCNs. For example, CT texture analysis is a tool used to assess the pathological variability in medical images [2, 3], which quantifies and normalizes the abstract texture features of lesions in CT images to identify the type of tumor and predict the patient's efficacy and prognosis [4–6]. At present, commonly used texture feature extraction methods for images include wavelet, LBP, HOG, GLCM, and Gabor [7]. In recent years, many new methods have emerged, such as the computer deep learning method—deep neural network (DNN) for the detection and identification of lesions on CT images [8, 9], which greatly improved the diagnostic efficiency of medical images. We used a new CT classification model of pancreatic serous cystic neoplasms and mucinous cystic neoplasms based on a DNN—MMRF-ResNet (Multi-channel-Multiclassifier-Random Forest-ResNet), which include 3 steps. Step 1: Image segmentation. (1) Obtain the single-channel manual outline ROI image. Two senior radiologists with 15 years of working experience manually outline the lesion in the axial CT image to obtain a single-channel manual outline ROI image, which serves the original CT grayscale ROI image. (2) Obtain the single-channel semi-automatic rectangular image. Based on the single-channel manual outline ROI image, the coordinates of the pixels on the top, bottom, left, and right of the single-channel manual outline ROI image are automatically recognized by the computer, and then this region is expanded 2 pixels outward to obtain the single-channel semi-automatic rectangular image that contains the pixels of the image of the complete lesion and its surrounding tissue. To some extent, a single-channel semi-automatic rectangular image can compensate for the limitation that the manual outline ROI image may not include the complete lesion area, which contains the pixels of the complete lesion and its surrounding tissue. Thus, this method provides a larger scope than the first method. (3) Construct a Multi-channel image. Convert the single-channel semi-automatic rectangular image into a Multi-channel CT image. The following operations are performed on the single-channel semi-automatic rectangular image during the conversion process: (a) adjust the window width and window level; (b) use the Canny operator (an image segmentation method based on edge recognition) to detect the image edges; (c) adjust the gradient magnitude to enhance the difference in image information between the lesion and the surrounding tissue. Step 2: Feature extraction. MMRF-ResNet classifier uses DNN ResNet to extract features of the Multi-channel image. ResNet uses 2048-dimensional feature output from the last layer of the network (Pooling layer, the role of which is to reduce the dimension by merging more than 2048-dimensional features) as the features of the lesion of each Multi-channel image. Step 3: Classification. The classification probabilities of three single classifiers (KNN classifier, Softmax classifier, and Bayes classifier) were integrated using the random forest classifier to distinguish between pancreatic SCNs and MCNs. The purpose of this study was to analyze the CT features of pancreatic SCNs and MCNs using the MMRF-ResNet model (The structure of MMRF-ResNet model is shown in Fig. 1) and to provide a better non-invasive imaging evaluation model for the identification of pancreatic SCNs and MCNs.

## Materials and methods

The ethics committee of the First Affiliated Hospital, Zhejiang University School of Medicine approved the study, and patients or their families provided signed informed consent. And all experiments were performed in accordance with relevant guidelines and regulations, research involving human research participants have been performed in accordance with the Declaration of Helsinki.

### Data

A retrospective analysis was performed using pancreatic unenhanced and contrast-enhanced CT images from 63 patients with pancreatic SCNs and 47 patients with MCNs (including three cases of mucinous cystadenocarcinoma) confirmed by pathology in the Department of Radiology, the First Affiliated Hospital, Zhejiang University School of Medicine, from December 2010 to August 2016. Among 110 patients, 68 patients accidentally found pancreatic lesions during physical examination without any clinical manifestations, 16 patients presented with abdominal distension, 20 patients presented with clinical manifestations of abdominal pain, and 6 patients presented with a sudden loss of weight. Before the operation, all 110 patients underwent CT examinations, but because the doctors could not confirm the nature of the pancreatic lesions in these patients before the operation, and they could not determine whether it was SCN or MCN, so all 110 patients underwent surgical resection of the lesions. The pathologist (J.Z., with 20 years of experience) were blinded to test results of computer deep learning but was provided with clinical information, CT images, and diagnostic reports. The following inclusion criteria were employed: (1) All patients underwent pancreatic enhancement three-phase CT scans on the same CT machine before surgery, including unenhanced, arterial and portal venous phases; (2) On CT cross-sectional images, lesions were displayed in at least 7 consecutive cross sections; (3) All surgically removed specimens and samples obtained by percutaneous puncture or endoscopic fine needle aspiration were examined by cytology or pathology. The exclusion criterion was individuals who are allergic to iodine contrast agents or those with severe renal insufficiency, and patients who had undergone radiation therapy or chemotherapy were excluded.

Images were acquired using a 256-slice spiral CT scanner (Brilliance iCT, Philips, Cleveland, OH, USA) using a pancreas standard scanning protocol (tube voltage 120 kV, tube current 300–550 mA). Scanning protocol: unenhanced scan, scanning range from dome to iliac crest, and layer thickness 3 mm. The arterial phase CT scan is performed 20 s after injection of contrast agent, and scanning ranged from 1 cm above the celiac artery to the third segment of the duodenum under conditions that can include the entire tumor, if we find that the size of the tumor exceeds the above range during the plain scan, the scan range of the arterial phase will be expanded to include the entire tumor, and the layer thickness is 3 mm. The portal vein CT scan is performed 60 s after the contrast agent is injected, the scan range and the layer thickness are the same as that of the arterial phase. CT enhanced scans were performed using 1.5 ml/kg iodinated contrast medium (Iohexol Injection, 300 mg I/ml, Ousu, Yangtze River Pharmaceutical Group) and 20 ml of saline injected through a peripheral vein at an injection rate of 3 ml/s with an automatic pump injector. Before the scan, water (750–1000 ml) was used as an oral contrast agent to fill the stomach cavity and duodenum.

### Lesion segmentation

Two methods are used to preprocess the image. Method one involves obtaining the single-channel manual outline region of interest (ROI) image. Two senior radiologists (F.C., with 15 years of experience and R.Y., with 15 years of experience) manually outline the lesion in the axial CT image (including the region where the tumor was present in all axial images) to obtain the single-channel manual outline ROI image (that is, the original CT grayscale ROI image). The radiologists were blinded to pathologic diagnosis but was provided with clinical information. Method two first involves extending the single-channel manual outline ROI image into a single-channel semi-automatic rectangular image via human–computer interaction (Yizhou Chen, with 7 years of experience and Kangjie Li, with 10 years of experience who were blinded to pathologic diagnosis and clinical information). The second step of method two involves constructing a Multi-channel image: The single-channel semi-automatic rectangular image is converted into a Multi-channel image. The following procedures are performed on the original image during the conversion process: (a) Adjust the window width and window level; (b) Use Canny operator (An image segmentation method based on edge recognition) to detect the edges of the lesions’ image; (c) Adjust the gradient magnitude to enhance the difference in image information between the lesion and the surrounding tissue.

### Statistical analysis

In this study, the following CT images including pancreatic cystic tumors were obtained: 4104 single-channel images, of which 2171 were SCNs and 1933 were MCNs; 4104 groups of Multi-channel images, including 2171 groups of SCNs and 1933 groups of MCNs (the number of single-channel images is the same as the number of groups of Multi-channel images). In each of the following steps, 80% of the CT images were used as the training data, and the remaining 20% were used as the test data randomly [10].

**Fig. 2:**
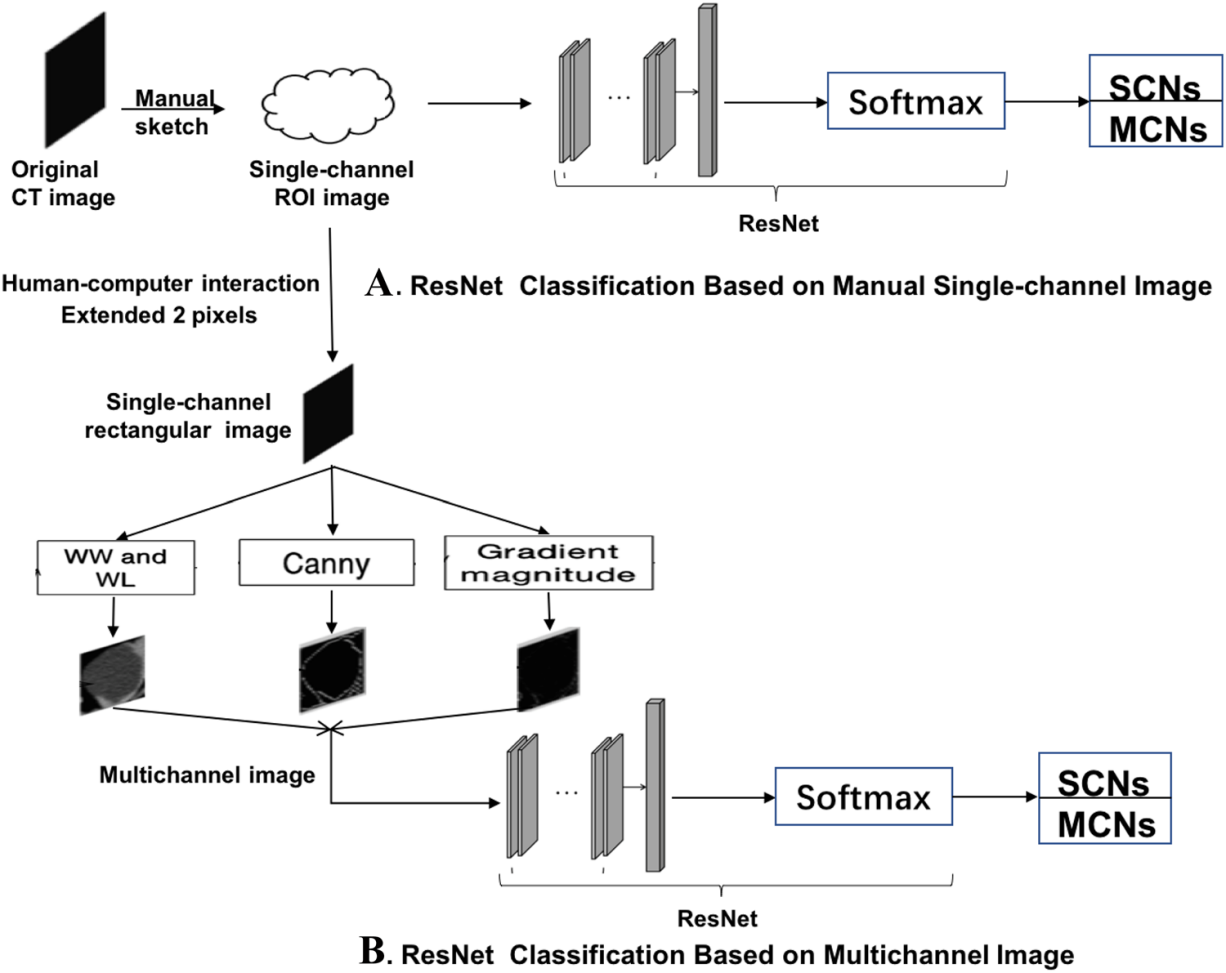
Classification model of pancreatic SCNs and MCNs based on single-channel and multichannel images. WW: Window Width, WL: Window Level, Canny: An efficient edge detection algorithm, Gradient magnitude: Magnitude of pixel gradient in the image. The gradient is a vector with direction and size. Softmax: a type of classifier

**Fig. 3:**
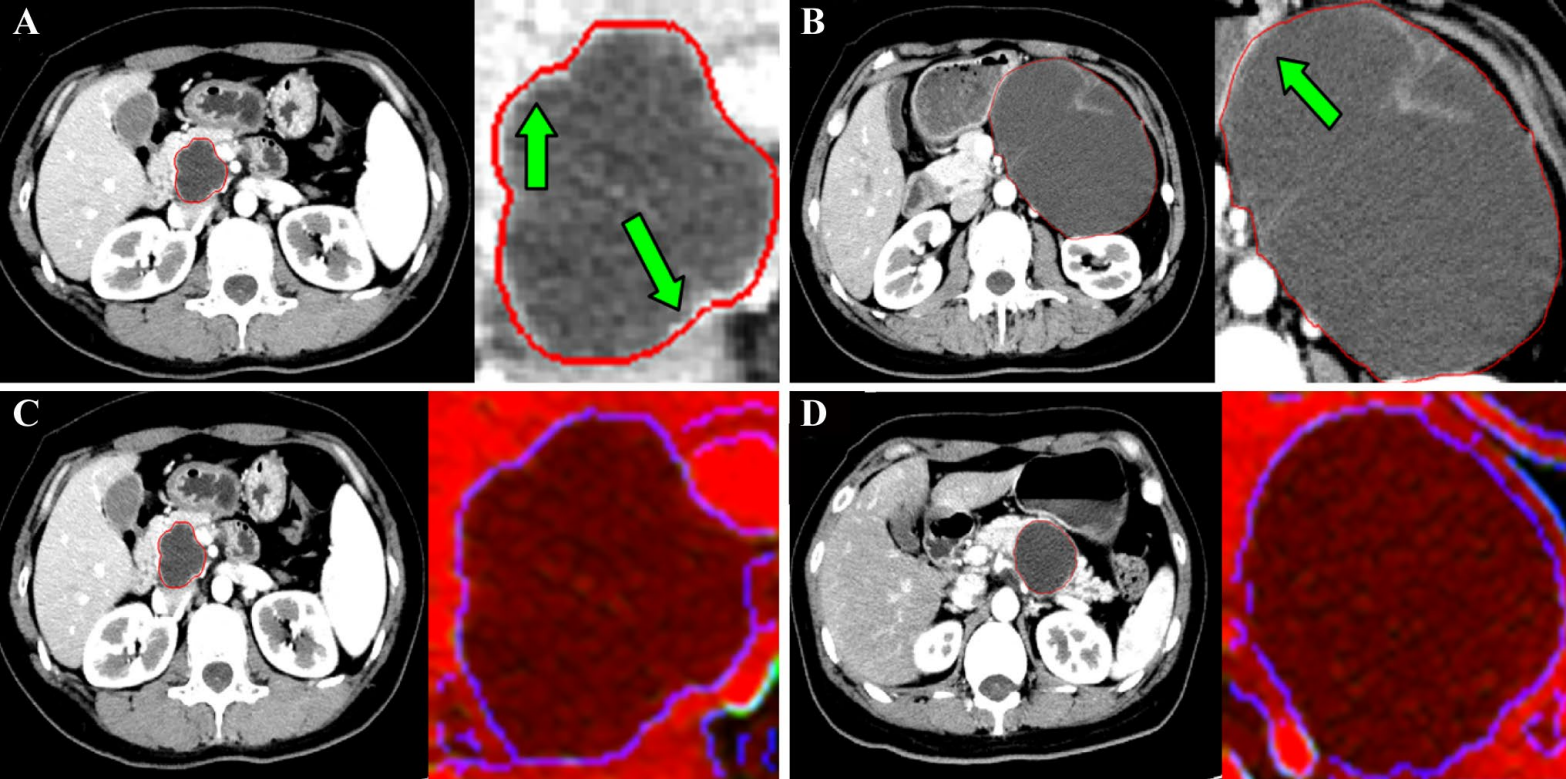
Single-channel manually outlined ROI and multichannel images. **A** and **C** ROI images of a 45-year-old woman with SCN. **A** Single-channel manually outlined ROI image. The lesion is manually outlined (the area within the red curve outline) inaccurately, causing erroneous information to be incorporated into the area of interest (the green arrow shows that part of the normal structure is included in the ROI). **C** Multichannel ROI image (area within the blue circle) of the same patient does not include the normal tissue area. **B** and **D** ROI images of a 56-year-old woman with MCN. **B** A single-channel manually outlined ROI image (the area within the red curve outline) shows that part of the normal structure is included (the green arrow). **D** Multichannel ROI image (area within the blue circle) of the same patient is more accurate than the single-channel manually outlined ROI image

The pancreatic tumor classification results were compared between two image preprocessing methods with the same feature extraction method and classifier to determine which image preprocessing method would achieve better classification efficacy for pancreatic SCNs and MCNs. (The structures of the single-channel manual outline model and Multi-channel model are shown in Fig. 2). Each CT image was processed by the above two preprocessing methods separately (examples of two preprocessed images are shown in Fig. 3). Then, ResNet was used to feature extraction, and the Softmax classifier was used to determine whether the lesion was SCN or MCN.

Different image feature extraction methods were compared. Using DNN ResNet and AlexNet, the features of each preprocessed Multi-channel image were extracted. Then, the Softmax classifier was used for the classification of pancreatic SCNs and MCNs. In addition, traditional image feature extraction methods (wavelet, LBP, HOG, GLCM, Gabor) and DNN ResNet were simultaneously employed to extract the features of each preprocessed Multi-channel image. Then, the SVM, KNN, and Bayes classifiers were used for the classification of pancreatic SCNs and MCNs.

Different classifiers were employed using the same image preprocessing method and the same feature extraction method to determine which classifier can achieve the best classification efficacy for pancreatic SCNs and MCNs. ResNet was used to extract the lesion features of all Multi-channel images. Then, single classifiers (KNN classifier, Softmax classifier, and Bayesian classifier) and multiple classifiers (majority voting rule method and random forest classifier) were used to classify pancreatic cystic tumors to determine the best classification strategy.

Based on the analysis results of the above three steps, the best model for CT classification of pancreatic SCNs and MCNs was determined (Fig. 1).

### Evaluation indicators

The following six indicators were used as evaluation indicators with pathological results serving as the gold standard: sensitivity, specificity, precision (number of true positives/(number of true positives + number of false positives) × 100%), accuracy, F1 score (an indicator used to measure the accuracy of the two-category model in statistics that takes into account the sensitivity and accuracy of the classification model, F1 score = 2 × precision × sensitivity/(precision + sensitivity), the maximum value of the F1 Score is 1, the minimum value is 0), and area under the receiver operating characteristic curve (AUC).

## Results

### Comparison of classification results of two image preprocessing methods for pancreatic SCNs and MCNs

Table 1 shows that the six indicators (precision, sensitivity, specificity, accuracy, F1 score, and AUC) using single-channel manual outline ROI images for the classification of pancreatic SCNs and MCNs are 83.03%, 86.69%, 81.64%, 84.17%, 84.78%, and 0.91. And the above six indicators using Multi-channel images are 92.58%, 92.58%, 92.31%, 92.45%, 92.58%, and 0.98. The single-channel manual outline ROI image may not be able to completely include the lesion area or may include the normal tissue area around the lesion (Fig. 3).

**Table 1:** Comparison of the results of classification of pancreatic SCNs and MCNs using Manual-ResNet and Multichannel-ResNet

Segmentation method of CT lesion image	Precision	Sensitivity	Specificity	Accuracy	F1 score	AUC	*p* value/Multi-channel
Manual	83.03%	86.69%	81.64%	84.17%	84.78%	0.91	< 0.001
Multichannel	92.58%	92.58%	92.31%	92.45%	92.58%	0.98	–

Manual: Manual outline of the lesion used to segment the lesion image, Multichannel: Semiautomatic segmentation of the lesion image using human–computer interaction for the manual image, ResNet: a type of DNN. ResNet extracts image features in the region of interest and then uses the Softmax classifier in the classification network to classify the lesions

### Comparison of different image feature extraction methods for the classification of pancreatic SCNs and MCNs

The six indicators (sensitivity, specificity, precision, accuracy, F1 score, and AUC) obtained using DNN ResNet are 92.58%, 92.58%, 92.31%, 92.45%, 92.58%, 0.97 when using Multi-channel images and the Softmax classifier for the classification of pancreatic SCNs and MCNs. And the above six indicators using AlexNet are 87.89%, 92.11%, 86.85%, 89.52%, 89.05%, 0.94 (Table 2). Moreover, it can be seen from Fig. 2 that the values of the six indicators obtained using ResNet to extract the image features and classify the lesions are all higher than using the currently commonly used feature extraction methods (wavelet, LBP, HOG, GLCM, and Gabor) when employing a Multi-channel image and the same classifier, including the SVM classifier, KNN classifier, or Bayes classifier.

**Table 2:** Comparison of classification results of pancreatic SCNs and MCNs by commonly used and DNN image feature extraction methods

Classification	Precision	Sensitivity	Specificity	Accuracy	F1 score	AUC	*p* value/Res_multi
AlexNet	87.89%	92.11%	86.85%	89.52%	89.05%	0.94	< 0.001
ResNet	92.58%	92.58%	92.31%	92.45%	92.58%	0.97	< 0.001
ResNet_SVM	93.17%	91.39%	93.05%	92.20%	92.27%	0.98	< 0.001
ResNet_KNN	97.26%	84.93%	97.52%	91.11%	90.68%	0.96	< 0.001
ResNet_Bayes	90.67%	91.63%	93.80%	92.69%	92.74%	0.94	< 0.001
*Wavelet* SVM	84.67%	80.62%	84.86%	82.70%	82.60%	0.91	< 0.001
*Wavelet* KNN	93.46%	68.42%	95.04%	81.49%	79.01%	0.92	< 0.001
*Wavelet* Bayes	68.85%	52.87%	75.19%	63.82%	59.81%	0.73	< 0.001
lbp_SVM	75.06%	73.44%	74.69%	74.06%	74.24%	0.80	< 0.001
lbp_KNN	78.02%	77.27%	77.42%	77.34%	77.64%	0.85	< 0.001
lbp_Bayes	56.60%	78.95%	37.22%	58.47%	65.93%	0.60	< 0.001
hog_SVM	83.25%	80.86%	83.13%	81.97%	82.04%	0.90	< 0.001
hog_KNN	86.02%	48.56%	91.81%	69.79%	62.08%	0.82	< 0.001
hog_Bayes	76.49%	55.26%	82.38%	68.57%	64.17%	0.75	< 0.001
glcm_SVM	47.06%	44.02%	48.64%	46.29%	45.49%	0.47	< 0.001
glcm_KNN	56.12%	55.98%	54.59%	55.30%	56.05%	0.58	< 0.001
glcm_Bayes	55.89%	93.06%	23.83%	59.07%	69.84%	0.64	< 0.001
gabor_SVM	66.75%	63.40%	67.25%	65.29%	65.03%	0.70	< 0.001
gabor_KNN	61.55%	72.01%	53.35%	62.85%	66.37%	0.65	< 0.001
gabor_Bayes	58.93%	71.05%	48.64%	60.05%	64.43%	0.60	< 0.001

ResNet and AlexNet: two types of classification methods based on deep neural networks used to extract image features of lesions and classify pancreatic lesions, Wavelet, LBP, HOG, GLCM, and Gabor: currently commonly used radiomics methods used for image extraction, SVM, KNN, and Bayes: classifiers used to classify lesions

### Comparison of single classifiers and multiclassifiers for the classification of pancreatic SCNs and MCNs

Table 3 demonstrated that the sensitivity of the KNN classifier (84.93%) is the worst of the three classifiers, but its specificity is the best (97.52%). The accuracy and F1 score obtained by the random forest classifier (92.69%, 92.74%) are greater than Softmax classifier (92.45%, 92.58%), KNN classifier (91.11%, 90.68%), and Bayes classifier (91.23%, 91.33%). The majority voting rule method was employed using Multi-channel images and ResNet for the classification of pancreatic SCNs and MCNs. The results for four evaluation indicators (precision, specificity, accuracy, and F1 score) obtained using the random forest classifier (MMRF-ResNet) which are 93.87%, 93.80%, 92.69%, 92.74% were higher than those obtained using the majority voting rule classifier (89.98%, 89.90%, 91.47%, 91.50%). However, the following order of AUC (from maximum to minimum) was obtained: Softmax classifier (0.97), MMRF-ResNet (0.96), KNN classifier (0.96), and Bayes classifier (0.94).

**Table 3:** Comparison of classification results of pancreatic SCNs and MCNs based on multiple classifiers

Classification	Precision	Sensitivity	Specificity	Accuracy	F1 score	AUC	*p* value/MMRF-ResNet
ResNet_Softmax	92.58%	92.58%	92.31%	92.45%	92.58%	0.97	< 0.001
ResNet_KNN	97.26%	84.93%	97.52%	91.11%	90.68%	0.96	< 0.001
ResNet_Bayes	90.67%	92.00%	90.46%	91.23%	91.33%	0.94	< 0.001
Majority voting	89.98%	93.09%	89.90%	91.47%	91.50%	0.96	< 0.001
MMRF-ResNet	93.87%	91.63%	93.80%	92.69%	92.74%	0.96	**–**

ResNet: a type of DNN method for extracting image features, Softmax, KNN, Bayes: classifiers, Majority voting: a type of multiple classifier that produces results consistent with the classification results of most classifiers, Random forest classifier: a type of multiple classifier that produces results consistent with the classification results of a higher-weight classifiers during the analysis of training data

## Discussion

At present, numerous challenges exist in the diagnosis and treatment of pancreatic SCNs and MCNs, such as the rational selection of imaging evaluation methods, the key points of correct imaging diagnosis, and conservative observation or surgical resection. SCNs are benign lesions that exhibit slow growth and a low malignant rate. Most of these lesions do not require surgery. However, if the tumor growth rate is significantly accelerated (diameter growth rate is greater than 2 cm/year) or signs of invasive growth are noted, surgical treatment should be considered [11]. MCNs have the potential to develop into pancreatic cancer, a recent review of 90 resected MCNs found that 10% of them contained either high-grade dysplasia or pancreatic cancer [12]. Therefore, it is necessary to seek an optimal strategy for the diagnosis of pancreatic SCNs and MCNs. Compared with CT and MRI examination, ultrasound examination of pancreatic lesions is susceptible to gastrointestinal gas, obesity, etc., and at contrast-enhanced ultrasound (CEUS) cystic tumors was correctly diagnosed with an sensitivity of 78.2% and with an NPV (negative predictive value) of 97.1%, which are much lower than that of CT examination [13]. Therefore, CT and MRI evaluation of the type of pancreatic SCNs and MCNs has become an important factor in determining treatment.

In the past, CT diagnosis of pancreatic SCNs and MCNs relied on the subjective empirical judgment of the radiologist [14], and sometimes they could use MR images for comprehensive analysis. Radiomic analysis methods eliminate the need for extensive clinical experience and avoid the interpretation of empirical imaging metrics [15]. However, at present, many radiomic methods rely on a large number of pre-defined features to quantitatively describe the characteristics of medical images, such as tumor volume and texture [16], and statistical methods are used to select the features most relevant to the results. Finally, the machine learning method is used to establish the diagnosis and prediction model. Among them, the commonly used methods include Logistic Regression, Support Vector Machine (SVM), and Random Forest [17]. Computer deep learning is a new field in machine learning research that can be used for medical image classification, segmentation, recognition, and brain function research [8, 9]. For example, DNN can make computers simulate the human brain for analytical learning and the human brain visual mechanism to automatically learn the abstract features of each level of data to better reflect the essential characteristics of the data. Thus, in this study, we used DNN to extract and classify the features of the lesions’ images and obtained the expected effect.

Image segmentation involves the extraction of ROI. This study first relied on an experienced radiologist to manually outline and segment the pancreatic cystic lesions in the original single-channel CT image. It is easy to produce volume effects when lesions are manually outlined inaccurately, causing erroneous information to be incorporated into the area of interest. Therefore, we preprocessed the single-channel manual outline ROI image to construct a Multi-channel image. During the conversion process, the window width, window level, and gradient magnitude are readjusted to enhance the difference in image information between the lesion and the surrounding tissue. Then, the Canny operator is used to obtain edge information of the lesion image, this effective edge detection method is based on the notion that the edge of the tumor is indicated by the local discontinuity of the image, such as sudden changes in grayscale, sudden changes in color, and sudden changes in texture structure [18]. Our study shows that six indicators (sensitivity, specificity, precision, accuracy, F1 score, and AUC) of classification effect using Multi-channel images are all better than using single-channel manual outline ROI image for the classification of pancreatic SCNs and MCNs. The Multi-channel image obtained after human–computer interaction and computer post-processing provides a more accurate lesion area image. Thus, Multi-channel CT images can better distinguish pancreatic SCNs from MCNs than single-channel manually outlined ROI images.

CNN (Convolutional Neural Network) is one type of DNN method [19] that extracts corresponding features in different layers, both AlexNet and ResNet represent excellent CNN methods [20, 21]. In this study, six indicators (sensitivity, specificity, precision, accuracy, F1 score, and AUC) of classification efficacy yielded better classification results with DNN ResNet than with AlexNet when using Multi-channel images and the Softmax classifier for the classification of pancreatic SCNs and MCNs with pathological results serving as the gold standard. The features extracted by ResNet were more representative than those extracted by the currently commonly used feature extraction methods (wavelet, LBP, HOG, GLCM, Gabor) for the classification of pancreatic SCNs and MCNs in the context of a multi-channel image and the same classifier, including the SVM classifier, KNN, and Bayes classifiers. ResNet performed better than AlexNet for the classification of pancreatic SCNs and MCNs. The following questions arise: Will the study draw the wrong conclusion if Softmax classifiers are exclusively used to classify lesions after extracting image features using ResNet and AlexNet? If the classification is performed by other classifiers, will the results change? The answer is no because when the features of different dimensions are entered into the same type of classifier for classification, the extracted features are the determining factor of the classification instead of the classifier. Alex's network structure contains fewer layers than ResNet, and the number of extracted features is far less than obtained with ResNet. Thus, ResNet improves the diagnostic efficacy of pancreatic SCNs and MCNs.

The role of the classifier is to learn the classification rules using the given and known training data categories and then classifying (or predicting) unknown data [22]. MMRF-ResNet uses a random forest classifier to integrate the classification probabilities of the KNN, Bayes, and Softmax classifiers. The random forest classifier is employed in this study because it can provide a certain classifier weight according to the classification result of a certain classifier during the analysis of the training data. Classifiers with better efficacy have higher weights, and classifiers with poorer efficacy have lower weights. The random forest classifier reasonably and comprehensively judges the classification results of the three classifiers and integrates the classification probabilities. Classification results of the majority voting rule method are consistent with the classification results of two or more classifiers among Softmax, KNN, and Bayes classifier. Thus, the results using the random forest classifier method (MMRF-ResNet) are more realistic than the results using the majority voting rule method.

There are still some shortcomings in this study. First, the study is retrospective, the number of samples is not large enough, and there are inherent limitations. Second, MMRF-ResNet requires that the lesion image is manually outlines for segmentation, and automatic segmentation is not possible. Third, we only analyzed the region of interest in images and did not analyze location information of the lesions (such as the head, body, and tail of the pancreas) and patient clinical information, such as gender, age, family history, and clinical symptoms, and the characteristics of the tumor have not been considered: size, grading, vascularization etc., for example are informations that can complete the clinical situation and they could be very useful notions. Fourth, we did not use the patient's MR examination images for comprehensive analysis. Fifth, radiomics features are affected by CT scanner parameters such as reconstruction kernel or section thickness, thus obscuring underlying biologically important texture features. This study did not use compensation methods to correct the variations of radiomic feature values caused by using different CT protocols [23]. Sixth, in the comparative study of single classifiers and multiclassifiers, given that the Softmax classifier uses more sampling points than the other three methods, the obtained AUCs ranked from the largest to the smallest are as follows: Softmax classifier, MMRF-ResNet, KNN classifier, and Bayes classifier. These results suggest that the diagnostic performance of MMRF-ResNet is lower than that of the Softmax classifier, but this result is inaccurate due to the number of sampling points. Finally, In how many cases the diagnosis match imaging findings and computed classification? The above shortcomings have yet to be further addressed.

In conclusion, in this study, Multi-channel CT images were obtained through preprocessing based on single-channel manual outline ROI images, and ResNet was used to extract CT image features of pancreatic SCNs and MCNs. The random forest classifier is used to integrate the classification probabilities of the KNN, Bayesian, and Softmax classifiers to determine the CT image properties of pancreatic SCNs and MCNs. Finally, a better classification result was obtained relative to the commonly used radiomics methods, suggesting that MMRF-ResNet is an ideal CT classification model for distinguishing between pancreatic SCNs and MCNs.

## Supplementary Information

Below is the link to the electronic supplementary material.

Supplementary file1 (DOCX 578 kb)

## Abbreviations

MMRF-ResNet: Multi-channel-Multiclassifier-Random Forest-ResNet
DNN: Deep neural network
SCNs: Serous cystic neoplasms
MCNs: Mucinous cystic neoplasms
AUC: Area under the receiver operating characteristic curve
