# Supplementary material for: CT classification model of pancreatic serous cystic neoplasms and mucinous cystic neoplasms based on a deep neural network
Source: Abdom Radiol (NY). 2021 Oct 12;47(1):232–41. doi: 10.1007/s00261-021-03230-5 (PMC8776667; doi:10.1007/s00261-021-03230-5)
Supplement: Supplementary file 1 — Supplementary file1 (DOCX 578 kb) [file 261_2021_3230_MOESM1_ESM.docx]

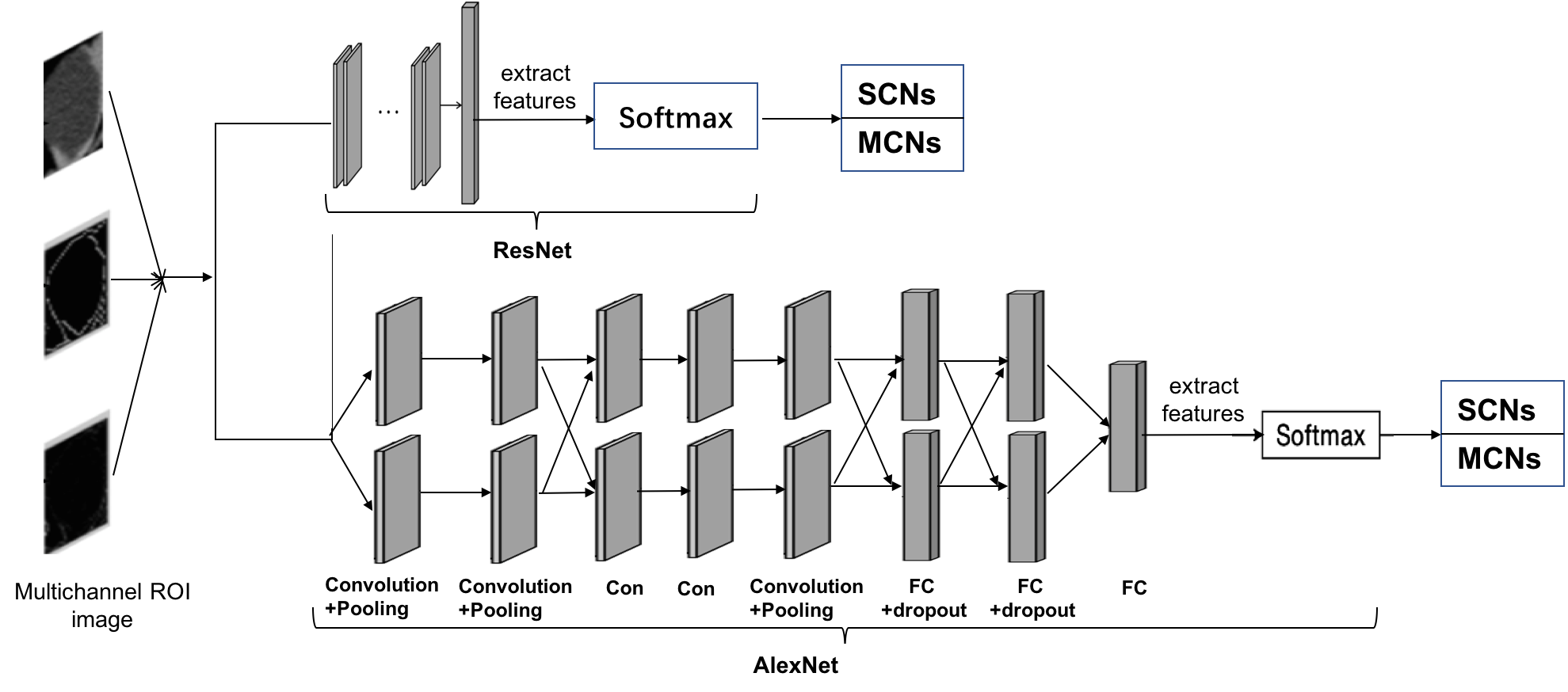


**Figure 4. Classification flowchart of pancreatic tumors based on feature extraction from AlexNet and ResNet networks.**

**Step 1**: Obtain a multichannel image of the lesion after preprocessing the original image. **Step 2**: Feature extraction of lesion image. Feature extraction method of DNN ResNet: Input the constructed multichannel image into ResNet for feature extraction. ResNet uses the 2048-dimensional features output from the last layer of the network (the pooling layer, the role of which is to reduce the dimension by merging more than 2048-dimensional features) to obtain the lesion features of each multichannel image. Feature extraction method of DNN AlexNet: Input the constructed multichannel image into AlexNet for feature extraction, and the features are output in 5 convolution layers and 3 fully connected layers and used as the lesion features of each multichannel image. **Step 3**: The Softmax classifier was used to classify pancreatic SCNs and MCNs. Con: Convolution layers, FC: Fully connected layers, Softmax: a type of classifier. Bayes: a type of classifier, AlexNet: a type of **deep neural network**, ResNet: a type of **deep neural network.**


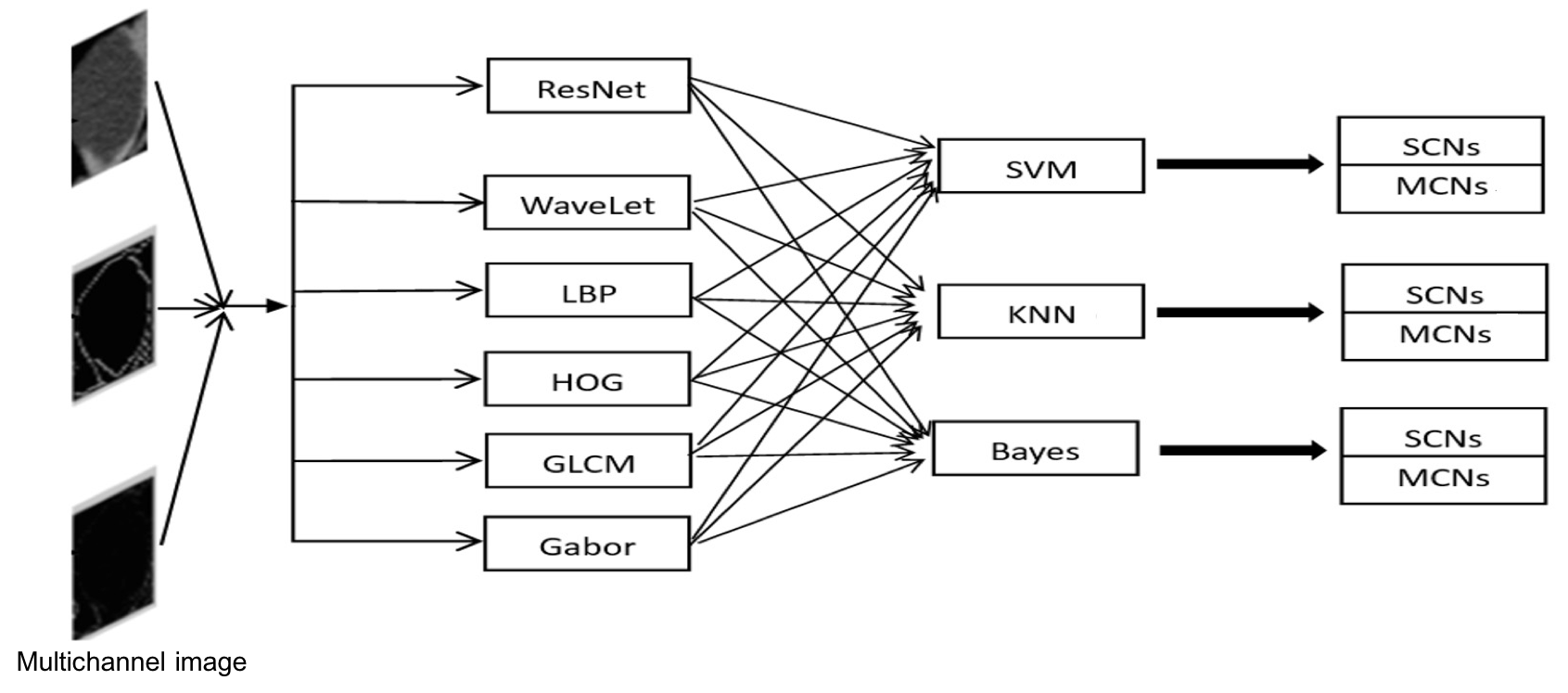


**Figure 5. Flow chart of pancreatic tumor classification based on ResNet and commonly used feature extraction methods.**

**Step 1**: Obtain a multichannel image of the lesion after preprocessing the original image. **Step 2**: Feature extraction. The ResNet network and **commonly used feature extraction methods (**wavelet, LBP, HOG, GLCM, and Gabor) are used to extract the features of multichannel images. **Step 3**: SVM, KNN and Bayes classifiers are used to classify pancreatic SCNs and MCNs. ResNet: a type of DNN, Wavelet, LBP, HOG, GLCM, and Gabor: Common feature extraction methods, SVM, KNN, Bayes: types of classifier**.**


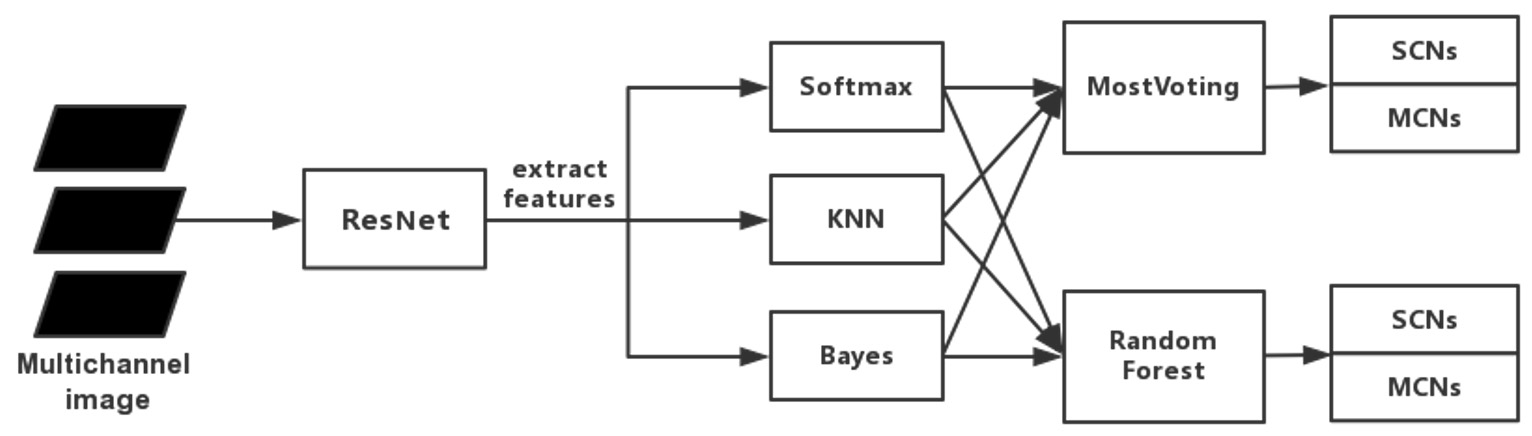


**Figure 6. Pancreatic tumor classification flowchart based on multiclassifiers.**

The features of the multichannel image are extracted by ResNet. Then, the features are input to the Softmax, KNN, and Bayes classifiers. Finally, the majority voting rule and random forest methods synthesize the classification results of the three classifiers to obtain the classification results for pancreatic SCNs and MCNs. ResNet: a type of DNN, Softmax, Bayes,KNN: types of classifier, Majority voting: a type of multiple classifier that produces results consistent with the classification results of most classifiers, Random forest classifier: a type of multiple classifier that produces results consistent with the classification results of higher-weight classifiers during the analysis of training data.
